# Decrease of larval and subsequent adult *Anopheles sergentii* populations following feeding of adult mosquitoes from *Bacillus sphaericus-*containing attractive sugar baits

**DOI:** 10.1186/s13071-015-0845-y

**Published:** 2015-04-23

**Authors:** Yosef Schlein, Günter C Müller

**Affiliations:** The Department of Microbiology and Molecular Genetics , IMRIC, Kuvin Centre for the Study of Infectious and Tropical Diseases, Faculty of Medicine, Hebrew University, Jerusalem, Israel

**Keywords:** Attractive-sugar- baits, *Bacillus sphaericus*, Mosquitoes larvae, Adults, *Anopheles sergentii*

## Abstract

**Background:**

*Bacillus sphaericus* is a mosquito-larvae pathogen which proliferates in the host cadavers, spreading and preserving the infection within the larval habitats for prolonged periods. In this pilot field study, we presented *B. sphaericus*-containing attractive sugar baits (ASB) to wild *Anopheles sergentii* adults, with the assumption that bait-fed, *B. sphaericus-*carrying mosquitoes are able to efficiently transmit the pathogen to the larval habitats, causing larval mortality and leading to a decrease in the subsequent adult population.

**Methods:**

The experiment was conducted over 75 days in two desert-surrounded streamlets. Blooming *Ochradenus baccatus* bushes were sprayed with bait solutions consisting of sugar and food dye marker solutions, with or without *B. sphaericus* at the experimental and control streamlets, respectively. Adult mosquito and larvae numbers were monitored before and after the treatment application, and the proportion of bait-fed adults was determined by visual inspection for dye presence.

**Results:**

Presence of food dye confirmed a large fraction of the adult mosquito population (70%-75%) readily ingested *Bacillus sphaericus*- containing bait*.* By the end of the study period, the larval population at the experimental site was six-fold smaller than the concurrent larval population at the control site. The ensuing adult *An. sergentii* population was also reduced to about 60% at the experimental site, while the adult mosquito population at the control site had increased 2.4 fold over the same time-frame. The reduction in adult mosquito numbers became apparent after a lag time (10 days), suggesting the treatment had minimal effect on adult longevity, also indicated by the post-treatment increase in the proportion of old mosquitoes and concomitant decrease in the proportion of young mosquitoes.

**Conclusions:**

Presentation of *B. sphaericus*-containing ASB substantially impacts the larval population, which in turn leads to a significant reduction of the ensuing numbers of adult mosquitoes. Although such outcomes may be the result of other causes, these preliminary results raise the possibility that adult mosquitoes can efficiently transmit the pathogen into the larval habitats. The transfer of *B. sphaericus* via contaminated adult mosquito carriers may allow introduction of pathogens to breeding places which are dispersed, hard to find, or difficult to access.

## Background

The spore-forming, aerobic bacterium, *Bacillus sphaericus* Neide produces highly-specific larvicidal compounds [1], and is relatively persistent in the larval habitats due to production of spores and toxins in larval cadavers, further perpetuating the infection within affected sites [2]. In this study we presented *B. sphaericus*-containing ASB to wild adult *Anopheles sergentii*, with the assumption that adult mosquitoes can efficiently transmit the pathogen to breeding locations*,* thus infecting such environments and cause substantial larval mortality. This hypothesis relies on the fact that a significant fraction of adult mosquitoes tends to congregate above breeding sites [3]. As the daily mortality of adult mosquitoes in the wild is relatively high, estimated to be in the range of 10% to 30% every 24 h [4], a fraction of the dying, pathogen-carrying adults are likely to fall into the aquatic environments which harbor the target larvae. This type of adults- to- larvae transmission of *B. sphaericus* was demonstrated earlier by Schlein and Pener [5] for *Culex pipiens* L, and in a later study by Devine et al. [6] which utilized adult *Aedes aegypti* as carriers of powdered juvenile hormone analogue to artificial larval habitats. The present study shows that application *B. sphaericus*-containing ASB caused substantial larval mortality, which in turn significantly reduced the subsequent adult population. In recent years, several studies have demonstrated the remarkable potential of attractive toxic sugar baits (ATSB) for controlling adult mosquito populations of the genera *Culex, Anopheles*, and *Aedes* [7-9]*.* The results of the present study suggest that in suitable combination, the addition of *B. sphaericus* to attractive toxic baits can enhance the efficiency such treatments by controlling both larvae and adult mosquito numbers.

## Methods

### Experimental sites

The lower Jordan Valley is a dry desert which contains several freshwater springs that are situated at the lower margins of the western mountains and drain into the northern part of the Dead Sea. In the dry season, the sole aquatic habitats consist of a trickle of water feeding a few sporadic puddles which harbor numerous *An. sergentii* larvae, with the surrounding vegetation serving as resting sites for adult mosquitoes. Two springs situated ca. 2 km apart were selected for the study sites. The watercourses at both the experimental and control sites are ca. 600 m long and 20-30 m in width, and are encompassed by dense thickets of vegetation in which reeds (*Phragmites australis* Clayton (Gramineae) are the dominant species, especially in shallow areas at the center of the sites. At the peripheries of the sites thickets of *Tamarix nilotica* Bunge (*Tamaricaceae)* and *Atriplex halimus* L. (Chenopodiaceae) (salt bush) are most abundant, while in the dry surrounding desert the sparse vegetation includes *Ochradenus baccatus* Delile (Resedaceae), *Nitraria retusa* Ascherson (Zygophyllaceae), *Suaeda aegyptiaca* and *Alhagi graecorum* Boiss*.*(Papilionaceae). As this study was conducted in the dry season, the only blossoms located in the vicinity of the *Anopheles sergentii* (Theobald) breeding sites belonged to highly-attractive *O. baccatus* bushes [7].

### Experimental design

The experiment was carried out in the dry season from the first of July to the 15th of September 2009. Mosquito monitoring began six days before the first and second treatments, which were performed on July 6th and August 8th, respectively.

The solution sprayed in the experimental site contained 1.5% W/V *Bacillus sphaericus* (serotype H5a5b, strain 2362; Valent BioSciences Corp., Libertyville IL, USA), 20% W/V sucrose, and 1.5% W/V red food- dye (Carmoisine E122, Stern, Natanya, Israel). Solution utilized in the control site did not include *B. sphaericus* and contained a different colored food dye (Food Blue No-1; Indigotin C.I., Stern, Natanya, Israel). Treatments were applied by spraying 0.5 liter of the suitable solutions to flowering *O. baccatus* bushes, known to be highly attractive to mosquitoes [6], at both the control site (n = 8 bushes) and the experimental site (n = 11 bushes), located at distances of 50-200 m from the water sources.

### Diurnal sampling of adults

Mosquitoes were collected with a sweep net on the fourth and fifth monitoring days prior to the treatments, and on two consecutive days one month after treatment. Sampling was performed between 7:00 and 9:00 AM, and was carried out in a similar manner and number of sweeps in both sites. Mosquitoes were anesthetized by introducing the net into a plastic bag containing a piece of cotton wool soaked with about 2 ml ethyl acetate.

### Nocturnal sampling of adults

Adults were trapped in each night pre- treatment and every third night afterwards. Trapping was conducted utilizing six UV-CDC- type traps in fixed locations at the outer perimeter of the vegetation of each site.

All specimens captured during diurnal and nocturnal sampling were investigated for the presence of food dye in the gut by visual inspection under a dissecting microscope.

### Diurnal sampling of larvae

Larvae were caught with a standard ladle between 8.00 and 9.00 AM, in shallow water heavily overgrown with reeds. Catching was performed at six fixed locations per site, with each sample consisting of six ladle-volumes stored in separate vials containing 70% ethanol.

### Age determination of adult mosquitoes

Random samples of 200 specimens of males or females mosquitoes which were caught diurnally by net were used for age assessments. The physiological age was determined for *An. sergentii* females by the Polovodova’s method [10], which determines the number of gonotrophic cycles by the number of ovarian dilatations. In addition, both sexes were examined for the presence of meconium (larval tissue residues) in the gut.

### Statistical analysis

Statistical analysis was conducted with SPSS software version 20.0 (SPSS inc., Chicago, IL). Larvae and adult mosquito counts were analyzed by performing a negative binomial analysis with a log link function to adjust for overdispersion of the data. Effects tested for significance included time, treatment type, and interaction between these two parameters. Wald chi square statistics were used to assess significance, which was taken at *P* < 0.05.

## Results

### Feeding of bait-solution by adult *An. sergentii*

Bait solutions were applied to plants which have been documented as important sugar-sources for desert-dwelling mosquito populations [6]. Indeed, assessment of food dye presence by visual inspection indicates *An. sergentii* adults frequently ingested the introduced bait solutions. Food dye marker was observed in 72.8% and 69.2% of the females captured nocturnally in the experimental and control sites, respectively. A slightly higher proportion of males were likewise found to have foraged on ABS solutions, with 75.0% and 72.4% of the specimens captured in the experimental and control sites, respectively, testing positive for the presence of food dye.

### Effect of the treatment on larval population numbers

**Figure 1:**
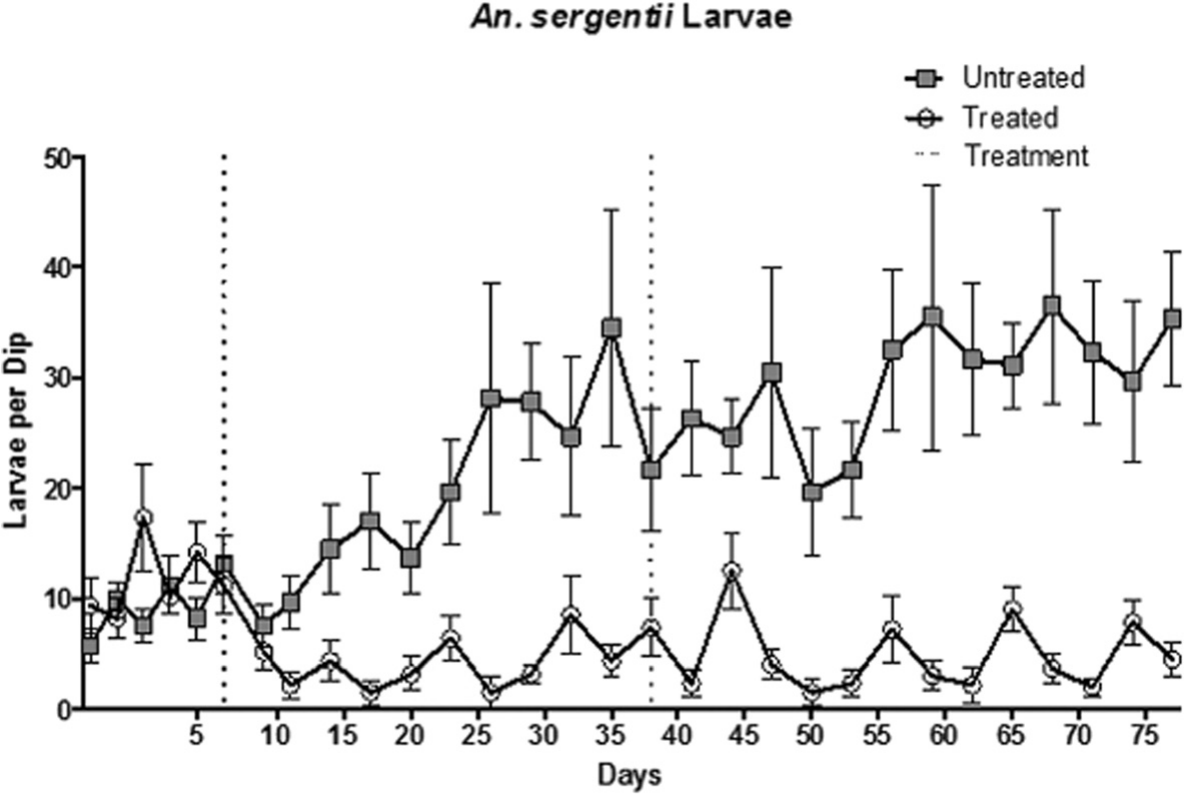
Number of *Anopheles sergentii* larvae per sample, each consisting of six ladle-volumes, collected at the control (squares) and experimental (circles) sites. The two applications of the ASB solution devoid of (control) or including *Bacillus sphaericus* (treatment) are indicated by the dotted lines.

The effects on the treatment on the larval population at the experimental were almost immediate. Larvae numbers declined by 56% within three days of the treatment application, and the mean catch remained significantly low thereafter, decreasing to 59.5% of the initial average for the remaining 45 days of the study (*P*<0.001; Figure 1). Moreover, the observed reduction of larval population in the experimental site does not convey the full magnitude of the treatment effect, since the observed decrease was apparently moderated by a concurrent positive population-growth trend. This can be inferred from the coincident increase in larvae numbers at the control area, which gradually increased almost four fold during the experimental period, reaching values which are 7 fold higher than those observed in the treatment site (Figure 1). The increasing difference between the larval populations of the control and the experimental sites over the study period suggests development of sub-optimal conditions in of the experimental site which are likely due to contamination of the larval habitat with *B. sphaericus*, transmitted by bait-fed adults, as no such differences were observed prior to the treatment. Application of a second treatment 38 days into the experiment had little impact on the larval population, possibly because a contamination of the larval habitat had already transpired.

### Effect of the treatment on adult mosquitoes

**Figure 2:**
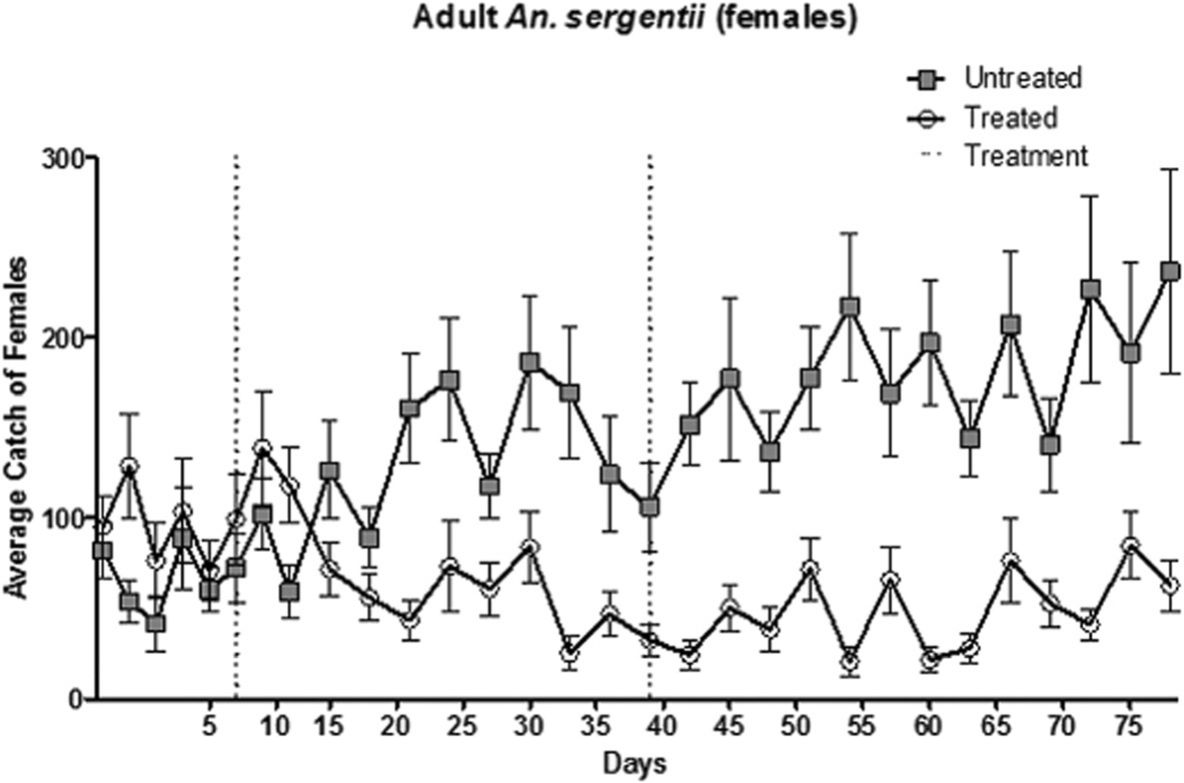
Number of adult female *Anopheles sergentii* captured with sweep-nets or UV-CDC traps at the control (squares) and experimental (circles) sites. The two applications of the ASB solution devoid of (control) or including *Bacillus sphaericus* (treatment) are indicated by the dotted lines.

In contrast with the immediate effects of the treatment on the larvae population, the relatively long lag time in treatment effect on the adult mosquitoes suggests *B. sphaericus* has little impact on their viability following ingestion of the bait. At both sites, no significant differences were found between the average number females captured pre-treatment and the average number of females captured 10 days following the treatment (*P* = 0.56; Figure 2). However, a significant decrease of 42% was observed 10 to 20 days after application of *B. sphaericus* bait at the treatment site (*P*˂0.001). In contrast, the number of females trapped in the control site increased significantly by 146% during the same timeframe (*P*˂0.001). Over the remaining period of the study, female numbers at the experimental site decreased by a further 7% of the initial values, while a 2.3 fold increase of female population numbers was observed at the control site.

**Figure 3:**
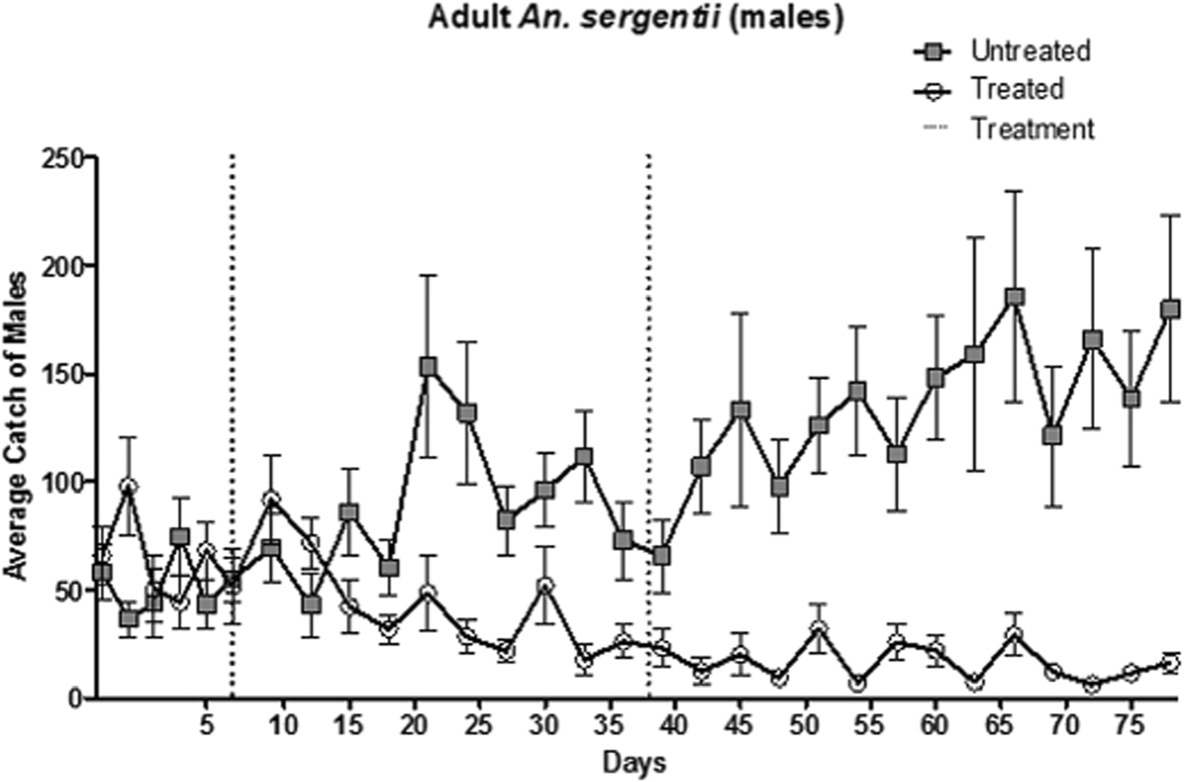
Number of adult male *Anopheles sergentii* captured with sweep-nets or UV-CDC traps at the control (squares) and experimental (circles) sites. The two applications of the ASB solution devoid of (control) or including *Bacillus sphaericus* (treatment) are indicated by the dotted lines.

Changes in male population numbers followed the same pattern (Figure 3). A similar average number of males were captured pre-treatment and 10 days after the treatment in the experimental area. The average number of males captured at the control site 10 days following the treatment increased slightly from pre-treatment values, albeit not significantly (*P* = 0.38). The mean number of males captured in the experimental site subsequently declined 10 to 20 days post treatment to 52% of the initial values, whereas at the control site the number of captured males significantly increased by 121% of the pre-treatment values (*P*<0.0001; Figure 3). Over the remaining study period, the male population at the experimental site declined further to 25% of the initial values, while at the control site the male population increased by 160%.

In line with the decline of larval population numbers at the experimental site, the proportion of nulliparous females (zero gonotrophic cycles) which were captured post-treatment was ca. 33% of the proportions of nulliparous females found in pre-treatment groups from both sites, as well as the proportion observed at control site post-treatment (Table 1)*.* Concomitantly, the proportion of old females (showing 5 or more gonotrophic cycles) at the experimental site increased by more than 3-fold following the treatment. In contrast, only a small increase in the proportion of old females was observed at the control site (from 12.5% pre-treatment to 14% post-treatment). A similar trend was also observed in the proportions of newly emerged male mosquitoes (e.g. one or two days old), identified by the presence of meconium tissue [11], the most apparent marker of young males. Meconium tissue was observed in 17.5% and 20.5% of the males captured pre-treatment at the experimental and control sites, respectively. This proportion remained similar in post-treatment samples captured at the control site (18.5%), while in samples captured at the experimental site the proportion of males with meconium decreased to 8.5% (Table 1). This pattern of decrease of adult population is a typical outcome of the use of a larvicide [12]. It is also noteworthy that an opposite trend is observed when utilizing vector-control methods that target adult mosquito populations, after which a decrease in the proportion of parous females and a concomitant increase in the proportion of nulliparous ones is observed [13], due to replenishing of the youngest group by newly emerged mosquitoes. These results further suggest that *B. sphaericus* has little impact on the viability of adult *An. sergentii*.

**Table 1:** **The proportions of different female** ***An. sergentii*** **age groups from the** ***bacillus sphaericus*** **treated experimental site and from the control site, where treatment did not include** ***B. sphaericus***

**Site & time**	**(%) with meconium**	% females* in groups classified by the numbers of gonotrophic cycles							
		0*	**1**	**2**	**3**	**4**	**5**	**6**	**>7**
**Control pre- treatment**	12.5	34.0	21.0	12.5	11.5	8.5	4.0	3.0	5.5
**Control post- treatment**	16.0	31.5	27.5	15.5	5.5	6.0	5.0	2.0	7.0
**Experimental pre-treatment**	15.0	32.5	23.5	18.5	8.0	7.5	2.5	3.5	4.0
**Experimental post-treatment**	5.5	13.0	16.0	14.0	20.5	12.0	8.5	6.5	9.5

Each of the examined four groups included 200 specimens. Physiological is age was defined by the number of gonotrophic cycles as indicated by ovarian dilatations, or by the presence of meconium tissue, a common marker of newly-emerged mosquitoes.

*Including specimens with meconium.

## Discussion

The aim of this study was to investigate a new approach to mosquito control by attractive sugar baits, which would complement the direct impact of insecticides on bait-fed adult mosquitoes. While such methods have been shown to be very effective in controlling adult mosquito numbers [13], one drawback of exclusively targeting adult populations is the constant replenishing of younger stages from maturing larvae and pupae, which in turn prolongs the treatment duration required to reduce the entomological inoculation rates below an acceptable threshold. Indeed, mosquito larvae develop in an extensive variety of natural and anthropogenic water habitats [2], which are often widely dispersed and difficult to locate, or constitute discrete sections of large, complex aquatic environments [14]. Specifically, lacking knowledge on larval ecology, and the wide variety of larval habitats which can be exploited by the primary vectors of malaria in the Afrotropical region - *Anopheles funestus* Giles and members of the *Anopheles gambiae* Giles complex, have discouraged efforts to develop and implement larval control strategies for these species [15]. Likewise, the main malaria vector in parts of southeast Asia, *An. dirus*, is generally exophilic, inhabiting forests where it breeds in many small, man-made, stagnant, transitory pools which are extremely difficult to find and destroy [16]. Other important vectors, such as *Aedes aegypti* and *Aedes Albopictus,* are also known to breed and oviposit in dispersed sites which are difficult to locate [17]. The current study highlights the potential advantages of utilizing adult mosquitoes for transmission of the larvicidal pathogen *B. sphaericus* to distant or secluded breeding sites.

It is reasonable to assume that the effect of *B. sphaericus* on the mosquito larvae population is dose dependent. Accordingly, adult mortality of *An. sergentii* in the field must be substantial for the efficient introduction of the pathogen into the larval habitat. This assumption is supported by previous observations which estimated the general mortality rate of mosquitoes in the wild in the ranges of ca. 10% to 40% every 24 h [4,18-20]. As adult female mosquitoes tend to congregate around water bodies in which they oviposit [3], it is probable the numbers of mosquitoes that die in-situ correlate with their relatively high densities. Considering the high proportion of bait-fed mosquitoes observed in this study (70%-75%), such natural mortality rates are likely sufficient for pathogen transmission. In a previous study [5], where only 47% of the female population was found to have ingested *B. sphaericus*-containing bait, transmission of *B. sphaericus* into the larval habitats was proven in laboratory studies by showing larvae collected pre-treatment were devoid of the*,* while those obtained following the treatment were positive for. In addition, dead, bait-fed adults which were provided to larvae had caused their subsequent mortality. Such bait-fed mosquito corpses were abundant in field samples obtained from the larval habitats following the application of the treatment [5].

While not directly verified, the immediate effect of the treatment on the larval population in this study suggests transmission of *B. sphaericus* by *An. sergentii* adults was quite effective, in view of the nearly identical environmental condition in the experimental and control sites, and the similar larval population densities which were recorded pre-treatment at both sites. Despite these compelling indications, it is possible the decrease in larval numbers at the experimental site may have been instigated by other factors. For example, adult mortality caused by contact or ingestion of *B. sphaericus* has been documented, along with a decrease in egg raft production by *Culex quinquefasciatus* in aqueous suspensions of *B. sphaericus* [21]. The results presented above suggest *B. sphaericus* had a little effect on adult *An. sergentii* longevity, as indicated by the lag in the impact of the treatment on the adult population (Figures 2 and 3), and the significantly higher proportion of parous females in samples captured post-treatment in experimental site (Table 1). Nonetheless, while mosquito mortality (and consequent decrease in oviposition) is unlikely to be the cause of the observed decline in larval number, the possibility of reduced fecundity due to physiological or behavioral changes of *B. sphaericus*-fed adult mosquitoes cannot be completely dismissed. Further experiments are required to unequivocally prove the infection-capacity of adults, as well as optimize the method to achieve higher infection rates.

A prerequisite of the described vector-control method is successful luring of adult mosquitoes to feed on the toxic bait despite the availability of competing sugar-sources. In our experiments, the blossoms of naturally-growing *Ochradenus baccatus* bushes in the control and experimental sites served as attractants to the baits, as these were shown in a previous study to be highly attractive to *An. sergentii* [7]. While the present study also reiterates these results, utilizing blossoms of local flora as attractants is limited by their seasonality and by dependence on their presence in suitable locations. Moreover, flowers that attract mosquitoes may be important sugar-sources of non-target insects which are thus also exposed to the toxin. Such insects are rare in mosquito congregation areas, near breeding sites or in resting sites, where the presence of other insects and their exposure to the ATSB toxin which is presented there is minimal [22]. Consequently, higher efficacy, specificity, and experimental flexibility can be gained by including specific mosquito attractants in ASB solutions [9,23,24]. This enables freedom in the choice of location and manner of bait presentation, either as a spray on the vegetation or bait stations.

## Conclusions

This pilot study demonstrates the plausibility of using the larvicide *Bacillus sphaericus* in attractive sugar baits. While commonly-tested ATSB methods which utilize insecticides are highly effective in controlling adult mosquito populations, their negligible impact on juvenile stages prolongs the duration required to achieve significant reduction of the adult population. Consequently, addition of larvicidal pathogens to commonly-used insecticides in ATSB solutions can significantly enhance the efficacy of such methods, by additionally impacting the larval populations and preventing the constant influx of newly emerging mosquitoes. One advantage of utilizing adult mosquitoes as carriers of the pathogen is their ability to reach highly dispersed breeding grounds which are difficult to locate. However, further experiments are required to unequivocally prove the infection-capacity of adults, as well as optimize the method to achieve maximal infection rates.

## References

[1] Charles J-F, Nielsen-LeRoux C, Delecluse A (1996). *Bacillus sphaericus* toxins: Molecular Biology and Mode of Action. Ann Rev Entomol.

[2] Floore TG (2006). Mosquito larval control practices: past and present. J Am Mosq Control Assoc.

[3] Le Menach A, McKenzie FE, Flahault A, Smith DL (2005). The unexpected importance of mosquito oviposition behavior for malaria: non-productive larval habitats can be sources for malaria transmission. Malaria J.

[4] Gillett JD (1971). Mosquitoes.

[5] Schlein Y, Pener H (1990). Bait-fed adult *Culex pipiens* carry the larvicide *Bacillus sphaericus* to the larval habitat. Med Vet Entomol.

[6] Devine GJ, Perea EZ, Killeen GF, Stancil JD, Clark SJ, Morrison AC (2009). Using adult mosquitoes to transfer insecticides to *Aedes aegypti* larval habitats. Proc Nat Acad Sci USA.

[7] Müller GC, Schlein Y (2006). Sugar questing mosquitoes in arid areas gather on scarce blossoms that can be used for control. Int J Parasitol.

[8] Müller GC, Junnila A, Schlein Y (2010). Effective Control of adult *Culex pipiens* by spraying an attractive toxic sugar bait solution in the vegetation near larval habitats. J Med Entomol.

[9] Müller GC, Schlein Y (2008). Efficacy of toxic sugar baits against adult cistern-dwelling *Anopheles claviger*. Trans Roy Soc Trop Med Hyg.

[10] Detinova TS (1962). Age grouping methods in Diptera of medical importance. Monogr Ser World Health Organ.

[11] Romoser WS, Moll RM, Moncayo AC, Lerdthusnee K (2000). The occurrence and fate of the meconium and meconial peritrophic membranes in pupal and adult mosquitoes (Diptera: Culicidae). J Med Entomol.

[12] Graham JE, Bradley IE (1972). Changes in the age structure of Culex pipiens fatigans Wiedemann population in Rangoon, Burma after intensive larviciding. J Med Ent.

[13] Müller GC, Beier JC, Traore SF, Toure MB, Traore MM, Bah S (2010). Successful field trial of attractive toxic sugar bait (ATSB) methods in Mali advances the search for viable new tools to locally control and eliminate malaria in Africa. Malaria J.

[14] Rejmanekova E, Savage HM, Rodriguez M, Roberts DR, Rejmanek M (1992). Aquatic Vegetation as a Basis for Classification of *Anopheles albimanus* Weideman (Diptera: Culicidae) Larval Habitats. Environ Entomol.

[15] Walker K, Lynch M (2007). Contributions of Anopheles larval control to malaria suppression in tropical Africa: review of achievements and potential. Med Vet Entomol.

[16] Pates H, Curtis C (2005). Mosquito behavior and vector control. Annu Rev Entomol.

[17] Chan KL, Ho BC, Chan YC (1971). *Aedes aegypti* (L.) and *Aedes albopictus* (Skuse) in Singapore City 2. Larval habitats. Bull Wld Hlth Org.

[18] McDonald PT (1977). Population characteristics of domestic *Aedes aegypti* (Diptera: Culicidae) in villages on Kenya coast. 1. Adult survival and population size. J Med Entomol.

[19] Muir LE, Kay BH (1998). *Aedes aegypti* survival and dispersal estimated by mark- release-recapture in northern Australia. Am J Trop Med Hyg.

[20] Laurence BR (1963). Natural mortality in two filarial vectors. Bull Wld Hlth Org.

[21] Zahiri NS, Mulla MS (2005). Non-larvicidal effects of *Bacillus thuringiensis israelensis* and *Bacillus sphaericus* on oviposition and adult mortality of *Culex quinquefasciatus* Say (Diptera: Culicidae). J Vector Ecol.

[22] Khallaayoune K, Qualls WA, Revay EE, Allan SA, Arheart KL, Kravchenko VD (2013). Attractive toxic sugar baits: control of mosquitoes with the low-risk active ingredient dinotefuran and potential impacts on nontarget organisms in Morocco. Environ Entomol.

[23] Müller GC, Kravchenko VD, Schlein Y (2008). Decline of *Anopheles sergentii* and *Aedes caspius* populations following presentation of attractive, toxic (Spinosad), sugar bait stations in an oasis. J Amer Mosq Control Assoc.

[24] Naranjo DP, Qualls WA, Müller GC, Samson DM, Roque D, Alimi T (2013). Evaluation of boric acid sugar baits against Aedes albopictus (Diptera: Culicidae) in tropical environments. Parasitol Res.

